# Plasmacytoid Dendritic Cells Are Inefficient in Activation of Human Regulatory T Cells

**DOI:** 10.1371/journal.pone.0044056

**Published:** 2012-08-29

**Authors:** Mario Hubo, Helmut Jonuleit

**Affiliations:** Department of Dermatology of the University Medical Center of the Johannes Gutenberg-University Mainz, Mainz, Germany; University of Southern California, United States of America

## Abstract

**Background:**

Dendritic cells (DC) play a key role in initiation and regulation of immune responses. Plasmacytoid DC (pDC), a small subset of DC, characterized as type-I interferon producing cells, are critically involved in anti-viral immune responses, but also mediate tolerance by induction of regulatory T cells (Treg). In this study, we compared the capacity of human pDC and conventional DC (cDC) to modulate T cell activity in presence of Foxp3^+^ Treg.

**Principal Findings:**

In coculture of T effector cells (Teff) and Treg, activated cDC overcome Treg anergy, abrogate their suppressive function and induce Teff proliferation. In contrast, pDC do not break Treg anergy but induce Teff proliferation even in coculture with Treg. Lack of Treg-mediated suppression is independent of proinflammatory cytokines like IFN-α, IL-1, IL-6 and TNF-α. Phenotyping of pDC-stimulated Treg reveals a reduced expression of Treg activation markers GARP and CTLA-4. Additional stimulation by anti-CD3 antibodies enhances surface expression of GARP and CTLA-4 on Treg and consequently reconstitutes their suppressive function, while increased costimulation with anti-CD28 antibodies is ineffective.

**Conclusions/Significance:**

Our data show that activated pDC induce Teff proliferation, but are insufficient for functional Treg activation and, therefore, allow expansion of Teff also in presence of Treg.

## Introduction

Human dendritic cells (DC) in peripheral blood form a heterogenous population composed of conventional DC (cDC), characterized as lineage^neg^ CD11c^+^ cells and lineage^neg^ CD123^+^ CD303^+^ CD304^+^ plasmacytoid DC (pDC) [1], [2]. DC function strongly depends on their activation state. Under steady state conditions, cDC exhibit an immature phenotype and are involved in maintenance of peripheral tolerance [2], [3]. The role of pDC during steady state still remains controversial. After specific activation, pDC are able to induce T cells with a regulatory phenotype [4]. Moreover, a population of human thymic pDC was suggested to drive the development of naturally occurring regulatory T cells (Treg) [5], [6], demonstrating the impact of pDC on peripheral T cell tolerance [7].

By suppressing T cell activation in absence of infectious agents, naturally occurring CD4^+^CD25^+^Foxp3^+^ Treg are critically involved in the homeostasis of a balanced immune system. Treg dysfunction often results in severe autoimmune diseases or even death [8]. The suppressive effector function of Treg is enabled after antigen specific activation via their T cell receptor and is antigen nonspecific [9]–[11]. Therefore, Treg function is strongly related to DC activation status [12]. It has been shown that activated cDC can induce Treg proliferation and expansion, thereby abrogating their anergic state, a typical Treg attribute [13], [14].

Once activated, cDC and pDC differ strongly in their T cell stimulatory capacity. While pDC have impaired stimulatory potential, activated cDC are potent T cell stimulators, as they express high levels of costimulatory molecules and cytokines [2]. Toll like receptor (TLR)-mediated recognition of pathogen associated molecular patterns plays a pivotal role in the activation process of DC. Conventional DC express TLR-2 to -6 and TLR-8, allowing responses against a broad range of bacterial or viral pathogens. Plasmacytoid DC on the other hand, only express TLR-7 and TLR-9, responsible for recognition of viral products and RNA/DNA/immunocomplexes. Therefore, together with the production of large amounts of type-I interferons (IFN) pDC are effective mediators of protective responses in anti-viral immunity [15].

Beside this protective role, pDC also exhibit pathogenic potential. For instance, their accumulation associates with different autoimmune diseases. In psoriasis patients, pDC migrate into the skin, where they sense self-DNA and produce type-I IFN thereby accelerating the disease. Further, it has been suggested that pDC promote the progression of systemic lupus erythematosus [16].

Thus, the role of DC in the network of immune responses is bifunctional. They act either immunogenic or have tolerogenic function. However, little is known regarding the capability of pDC to modulate the crosstalk of Treg and Teff. Therefore, we investigated the functional properties of pDC as antigen-presenting cells for Treg and Teff. We show that human pDC can activate Teff, but they are inefficient activators of Treg with the consequence of Teff proliferation in presence of potentially suppressive Treg.

## Materials and Methods

### Culture Medium and Antibodies

DC and T cells were cultured in X-VIVO-15 (Lonza, Belgium). Flow cytometric analysis was performed using the following antibodies. Rat IgG: anti-HLA-DR (YD1/63.4.10, Serotec), anti-GARP (G14D9, eBioscience), mouse IgG: anti-CD3 (UCHT1, BD Biosciences), anti-CD4 (RPAT-4, BD Pharmingen), anti-CD11c (B-ly6; BD Biosciences), anti-CD14 (M5E2, BD Biosciences), anti-CD19 (HIB19, BD Biosciences), anti-CD40 (5C3, BD Biosciences), anti-CD45RO (UCHL1, BD Biosciences), anti-CD58 (AICD85, Immunotech), anti-CD80 (MAB104, Beckman Coulter), anti-CD86 (BU63, Serotec), anti-CD123 (9F5, BD Biosciences), anti-CD127 (HIL-7R-M21, BD Pharmingen), anti-CD303 (AC144, Miltenyi Biotec), anti-Foxp3 (259D, eBioscience), anti-CTLA-4 (BNI3, BD Biosciences). Cell viability was determined using 7-AAD (eBioscience). Stained cells were measured on LSRII with FACS Diva Software (BD Biosciences).

**Figure 1:**
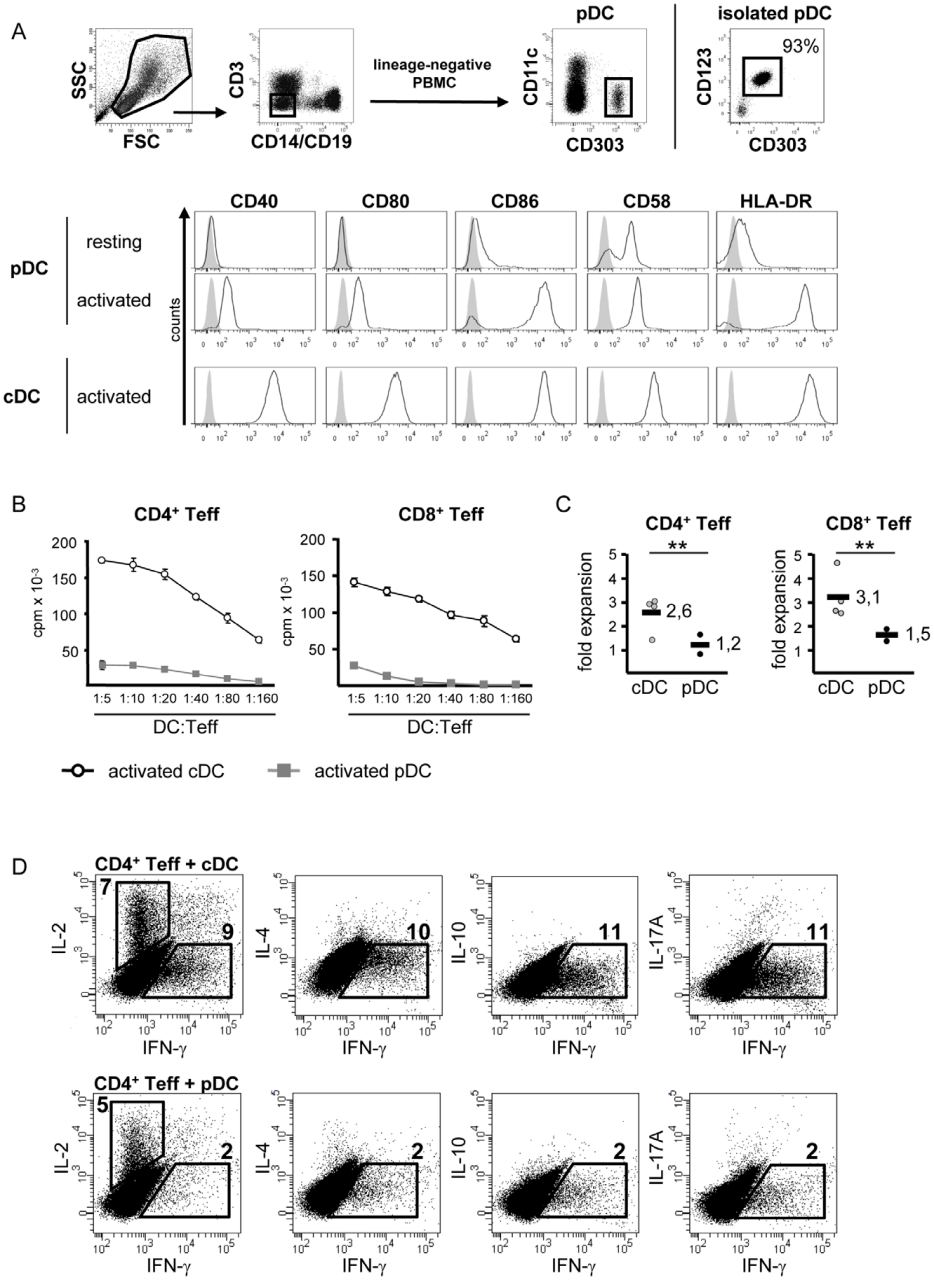
Plasmacytoid dendritic cells show weak T cell stimulatory capacity. A: Flow cytometric analysis of human PBMC and DC. pDC are characterized as lineage^−^ CD11c^−^ CD303^+^ cells (middle panel). Dot plots (right side) exemplarily depicts isolated pDC, percentage indicates purity. Histograms show surface expression of CD40, CD80, CD86, CD58 and HLA-DR on resting and CpG/IL-3-activated pDC and cytokine cocktail activated cDC. Isotype-matched control mAb staining is shown in grey. One representative result of three independent experiments is shown. B: T cell stimulatory capacity of activated pDC and cDC. CD4^+^ or CD8^+^ Teff were stimulated with allogeneic pDC or cDC at different DC:Teff ratios. Teff proliferation was determined on day 4 of culture by incorporation of ^3^H-Tdr and presented as mean ± SD of triplicates. One representative experiment out of 3 is shown. C: Expansion of DC-stimulated Teff. Expansion rates of CD4^+^ or CD8^+^ Teff after stimulation with allogeneic cDC (n = 4) or pDC (n = 2), determined at day seven. The figure shows the relative fold expansion of initially stimulated Teff. Bar indicates mean values. D: Cytokine profiles of Teff seven days after stimulation with pDC or cDC. Numbers represent the percentage of cytokine-positive Teff. One typical result out of six is shown. ** p<0.01.

### Generation of cDC and Isolation of pDC

cDC were generated from buffy coats of healthy volunteers as described previously [3]. In brief, PBMC were isolated by Ficoll density gradient centrifugation and monocytes were isolated by plastic adherence and cultured in X-VIVO-15 supplemented with 1% heat-inactivated autologous plasma together with 800 IU/ml GM-CSF (Leukine, Berlex) and 100 IU/ml IL-4 (CellGenix). Fresh media with GM-CSF (1600 U/ml) and IL-4 (100 IU/ml) was given at day 2 and day 4. Nonadherent cells were rinsed off at day 6, washed in PBS and 8×10^5^ cells per well were transferred to new 6-well plates (Corning Costar) in 3 ml culture media. For maturation, media was supplemented with IL-1β (10 ng/ml), TNF-α (10 ng/ml), IL-6 (1000 U/ml) (all from Strathmann Biotech) and 1 µg/ml PGE_2_ (Minprostin, Pharmacia & Upjohn). Terminal differentiated conventional DC were harvested on day 8 and used for T cell stimulation.

**Figure 2:**
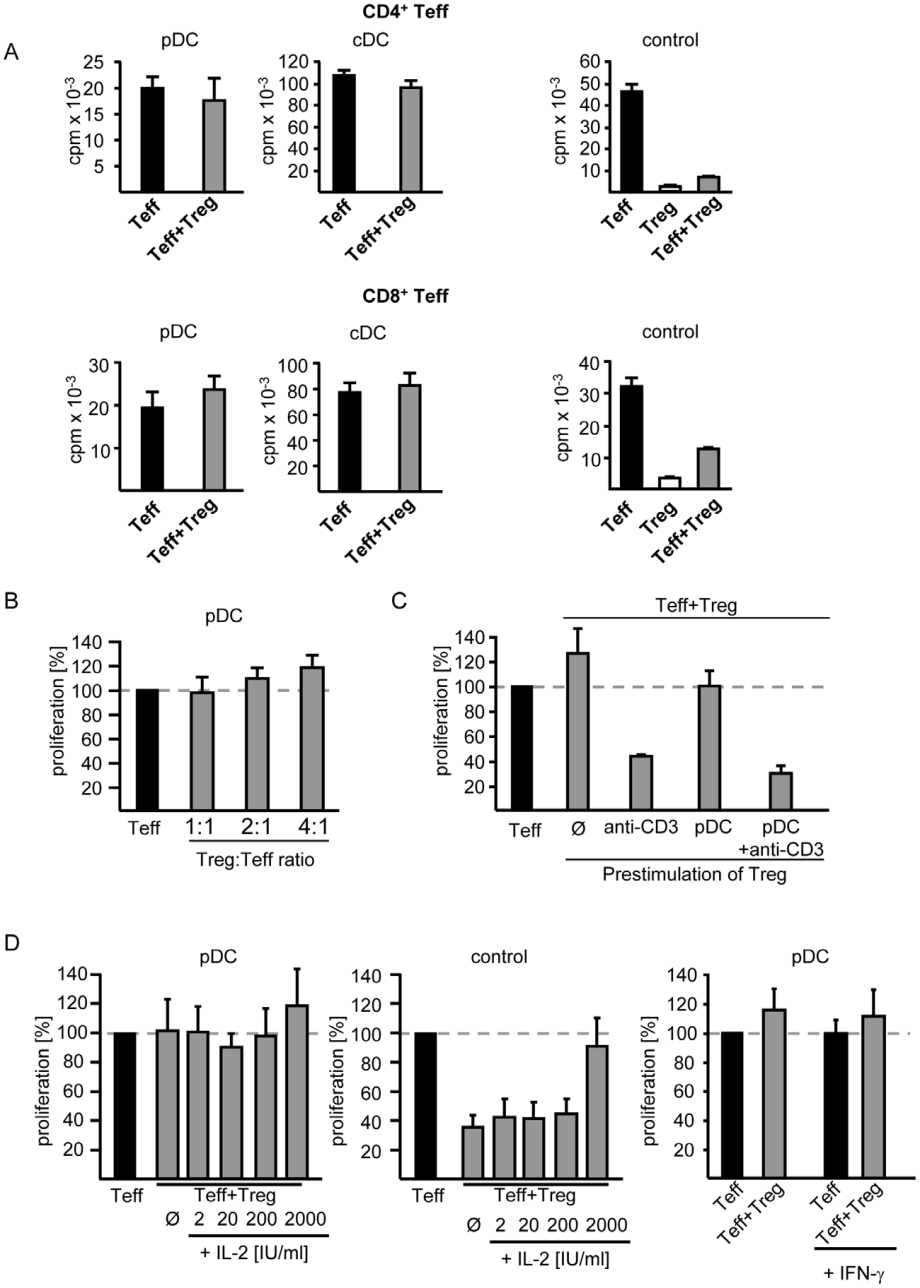
T effector cell proliferation induced by plasmacytoid dendritic cells in presence of regulatory T cells. A: Absence of Treg function in DC-stimulated cocultures with Teff. CD4^+^ (upper panel) or CD8^+^ (lower panel) Teff alone or in coculture with Treg (Treg:Teff 1∶1) from the same donor were stimulated with activated allogeneic pDC or cDC (left and middle panel). Polyclonal stimulation served as control for validation of Treg function (right panel). Proliferation was assessed on day 4 of culture by incorporation of ^3^H-Tdr and is depicted as mean ± SD of triplicates. One representative result out of 44 (pDC) respectively 9 (cDC) independent experiments is shown. B: Increased Treg frequencies in cocultures. CD4^+^ Teff were stimulated with activated pDC in presence of increasing Treg numbers. Mean values of three independent experiments are shown as proliferation of cocultures normalized to CD4^+^ Teff alone (set to 100%). C: Prestimulation of Treg. Treg were prestimulated with anti-CD3 mAb, allogeneic pDC or pDC plus anti-CD3 mAb for 20 h prior their use in suppression assays with syngenic CD4^+^ Teff (Teff:Treg 1∶1) or left untreated. T cell proliferation was assessed on day 4. Proliferation in cocultures was normalized to Teff proliferation in absence of Treg (set to 100%). D: Addition of exogenous IL-2 and IFN-γ to suppression assays. Cocultures (Teff:Treg 1∶1) were stimulated with allogeneic pDC (Teff/Treg:pDC 5∶1) in absence or presence of IL-2 or IFN-γ. Polyclonal stimulation served as control. Proliferation was analyzed on day 4 of culture by ^3^H-Tdr incorporation, presented as normalized to Teff proliferation in absence of Treg (set to 100%). Diagrams show mean values (± SD) of three independent experiments.

Plasmacytoid DC were isolated from PBMC with anti-CD304 Microbeads (1 µl/10^7^ PBMC, Miltenyi Biotec GmbH, Germany) according manufacturers indication. Purity of CD303^+^ CD123^+^ pDC was routinely >95%. For activation, pDC were cultured in X-VIVO-15 for 24 hours in 96-well plates (Corning Costar) at 10^5^ cells per well together with 10 ng/ml rhIL-3 (R&D Systems) and 1 µM CpG 2006 (TIB MOLBIOL, Germany).

### Isolation of T Effector Cells and Regulatory T Cells

Teff and Treg were isolated from buffy coats of healthy volunteers as described before [10], [17]. Briefly, CD25^+^ cells were separated using limited amounts of CD25-microbeads (Miltenyi Biotec) resulting in CD25^high^ cells. Afterwards, contaminations of CD25^+^ non T cells were depleted using CD14-, CD8-, and CD19-Dynabeads (Invitrogen/Dynal, Norway), resulting in a purity of CD4^+^CD25^high^Foxp3^+^ T cells of >90% (see Fig. 5a). Treg functionality was controlled in standard suppression assays [10].

**Figure 3:**
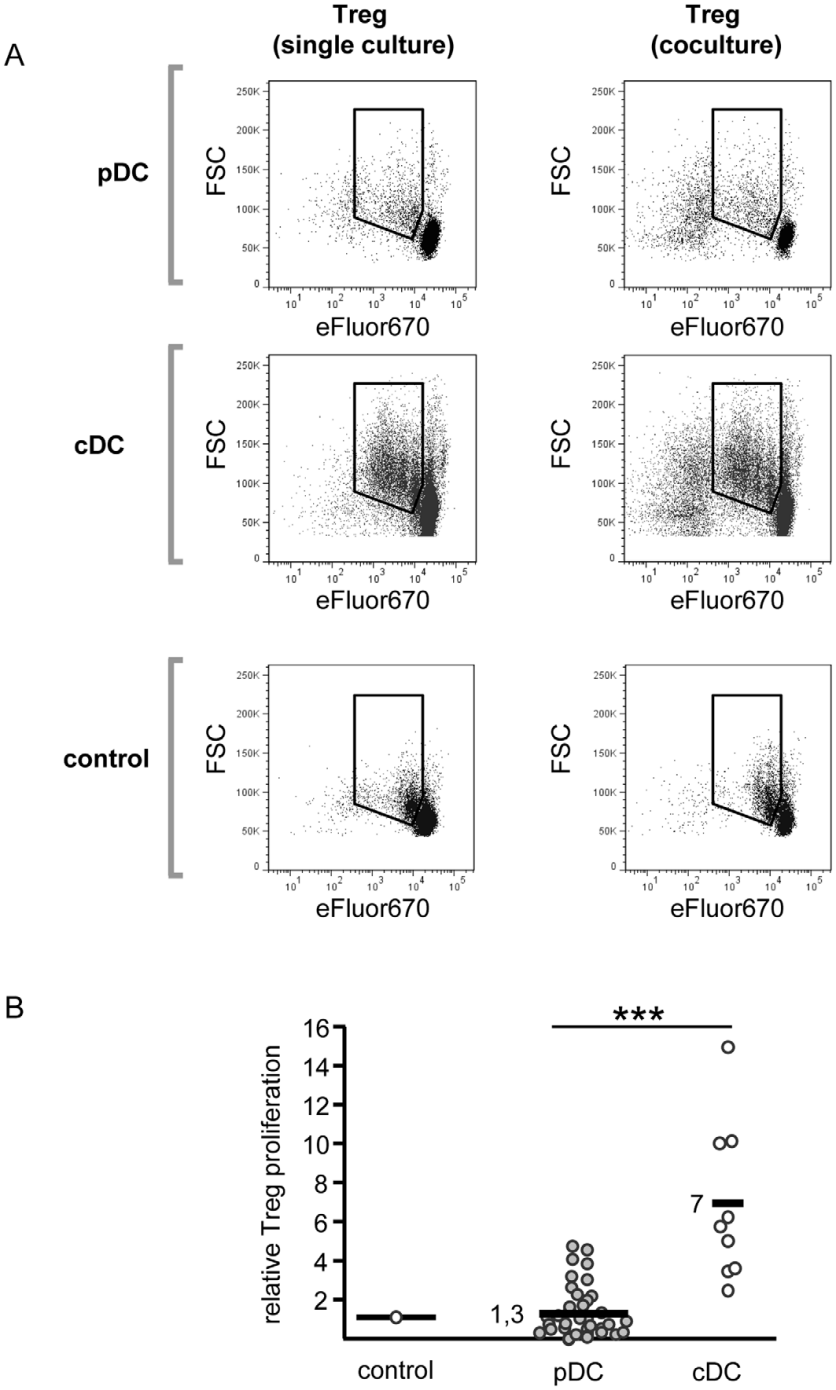
Plasmacytoid dendritic cells are unable to induce proliferation of regulatory T cells. A: Proliferation of DC-stimulated Treg alone or in coculture with Teff. eFluor®670-labeled Treg were stimulated alone (left panel) or in coculture with CD4^+^ Teff (ratio 1∶1, right panel). Allogeneic pDC or cDC (Treg/Teff:DC 5∶1) were used for stimulation, stimulation with anti-CD3 mAb plus irradiated T cell-depleted PBMC (standard culture) served as control. Proliferation was determined on day 4 of culture by flow cytometrical analysis of eFluor®670 dilution. One representative result of three independent experiments is shown. B: Treg proliferation induced by allogeneic pDC versus cDC. Treg were cocultured at a Treg:DC ratio of 5∶1 with either pDC or cDC. Treg stimulated with anti-CD3 mAb plus T cell-depleted PBMC served as control. Treg proliferation was determined by ^3^H-Tdr incorporation at day 4 of culture. Diagram shows Treg proliferation normalized to proliferation in standard culture (pDC: n = 43; cDC: n = 9) ** p<0.01.

CD4^+^ and CD8^+^ Teff were isolated using CD4/CD8-microbeads (Miltenyi Biotec); afterwards, CD25^+^ T cells were depleted with CD25-Dynabeads (purity: CD4^+^CD25^−^ or CD8^+^CD25^−^ Teff greater than 98%).

### T Effector Cell Stimulation

Stimulation of Teff was carried out in 96-well flat bottom “half area” plates (Corning Costar). For determination of DC-mediated Teff stimulation, 5×10^4^ Teff were cocultured with decreasing ratios of cDC or pDC. Suppression assays were accomplished by coculture of 5×10^4^ Teff with syngenic Treg at different ratios (Treg:Teff 4∶1-1∶1) or without Treg and stimulated with 10^4^ allogeneic pDC or cDC. In some experiments anti-CD3 mAb (0.5 µg/ml OKT-3), anti-CD28 (1 µg/ml CD28.2, BD Biosciences) or anti-CD40L (10 µg/ml, TRAP1, BD Biosciences) was used as an additional stimulus. If indicated, cultures were supplemented with different concentrations of IL-2 (Proleukin) or IFN-γ. Cultures stimulated with 0.5 µg/ml anti-CD3 mAb (OKT-3) together with 1.5×10^5^ irradiated (90 Gy) T cell-depleted PBMC (0.5 CD3-Dynabeads/cell; Invitrogen) served as positive control. Preactivation culture of isolated Treg: For some experiments 5×10^4^ Treg were precultured with 5 IU/ml IL-2 (Proleukin) or 0.5 µg/ml anti-CD3 (OKT-3) plus IL-2, or activated allogeneic pDC (1∶5, Treg:pDC). After 20 h these preactivated Treg were used in suppressor assays with CD4^+^ Teff (syngenic to Treg). For blockade of cytokines, cultures were supplemented with neutralizing antibodies against IL-1β (10 µg/ml, MAB201, R&D Systems), IL-6 (10 µg/ml, MAB206, R&D Systems), TNF-α (10 µg/ml, AB-210-NA, R&D Systems) or IFN-α (10 µg/ml, polyclonal antibody, PBL Interferon source). Proliferation was determined on day 4 of culture by additional 16 h pulse with 37 kBq/well ^3^H-Tdr.

**Figure 4:**
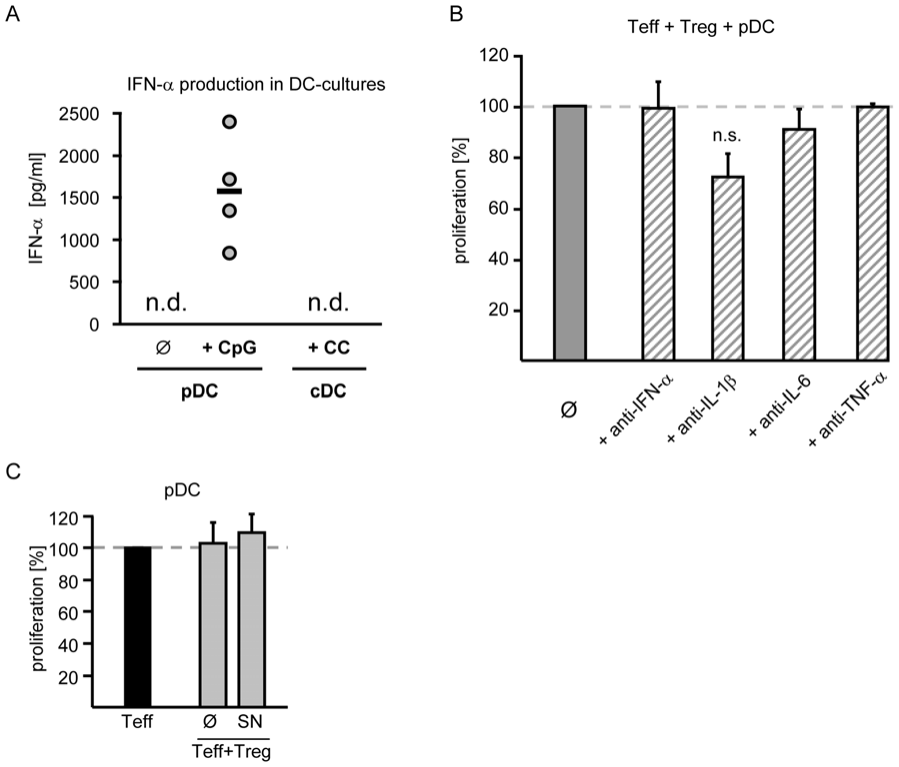
Neutralization of proinflammatory cytokines does not restore Treg suppression. A: Production of IFN-α by activated pDC. Supernatants of activated pDC (CpG/IL-3) or cDC (cytokine cocktail) cultures were harvested. Production of IFN-α was investigated by ELISA. Each circle resembles one individual experiment, bar represents mean; n.d.: not detectable, n = 4. B: Neutralization of cytokines in cocultures of Teff, Treg and pDC. Teff plus Treg (1∶1) were stimulated with allogeneic pDC at a ratio of 1∶5 in absence (Ø) or presence of neutralizing antibodies against IFN-α, IL-1β, IL-6 or TNF-α (10 µg/ml each). Proliferation in cocultures (mean ± SD of triplicates) is normalized to untreated cocultures (set to 100%). One representative experiment out of three is shown. C: Addition of supernatants from standard cultures. CD4^+^ Teff and Treg (1∶1) were stimulated with anti-CD3 mAb (0.5 µg/ml) plus irradiated TC-depleted PBMC. Supernatants from these cocultures were collected on day 3 and titrated (1∶2) to pDC-stimulated CD4^+^ Teff, Treg or cocultures. Proliferation was assessed on day 3 by ^3^H-Tdr-incorporation. Diagrams show mean values (± SD) of three independent experiments. Proliferation of untreated and treated cocultures was normalized to untreated or treated Teff-proliferation (set to 100%).

### Flow Cytometric Analysis of Proliferation

Freshly isolated Treg were washed in warm PBS, stained with 1 µM cell proliferation dye eFluor®670 (eBioscience) in PBS and cocultured with syngenic CD4^+^ Teff (ratio 1∶1) in presence of 10^4^ allogeneic pDC, cDC or with 0.5 µg/ml anti-CD3 mAb (OKT-3) and irradiated (90 Gy) T cell-depleted PBMC as control. Proliferation of viable (7-AAD-neg.) Treg was assessed on day 3 and 4 by analysis of eFluor®670 dilution. Flow cytometry was performed on LSRII (BD Biosciences) data were analyzed using FlowJo software (Tree Star).

**Figure 5:**
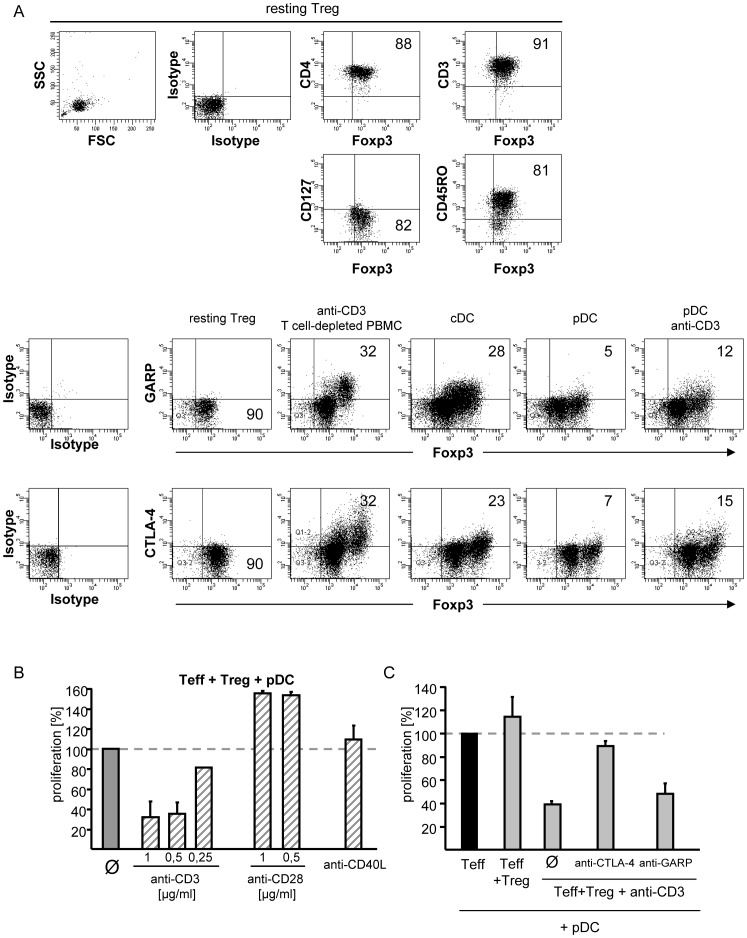
Plasmacytoid dendritic cells are unable to activate the suppressive function in human regulatory T cells. A: Surface expression of CTLA-4 and GARP on resting and differently stimulated Treg. Upper panels: flow cytometric characterization of resting CD25^+^ Tregs stained for indicated markers. Lower panels: Treg were stimulated with anti-CD3 mAb (5 µg/ml) plus T cell-depleted PBMC or allogeneic cDC (1∶5), pDC (1∶5) or pDC plus 5 µg/ml anti-CD3 mAb for 48 h. Flow cytometric analysis of CTLA-4 (extracellular expression), GARP and Foxp3 is shown. Percentages of the indicated surface marker on Foxp3^+^ Treg are displayed. One representative result out of four is shown. B: Additional anti-CD3 mAb stimulation restored Treg function in cocultures with Teff and pDC. CD4^+^ Teff cocultured with Treg (Treg:Teff 1∶1) were stimulated with allogeneic pDC alone or in presence of additional anti-CD3 mAb or anti-CD28 mAb in different concentrations or plate bound anti-CD40L (10 µg/ml). Proliferation (mean ± SD of triplicates) was analyzed on day 4 by ^3^H-Tdr incorporation and normalized to untreated cocultures (set to 100%). One representative result of three is shown. C: Blockade of CTLA-4 and GARP in pDC/anti-CD3-stimulated cocultures. CD4^+^ Teff cocultured with Treg (Treg:Teff 1∶1), stimulated with allogeneic pDC plus anti-CD3 mAb (0.5 µg/ml). Where indicated, anti-CTLA-4 mAb or anti-GARP mAb (10 µg/ml each) were added. Proliferation (mean ± SD of triplicates) was analyzed on day 4 and normalized to untreated cocultures (set to 100%). Results are shown as mean proliferation (± SD) of three independent experiments.

### Cytokine Analysis

10^6^ CD4^+^CD25^−^ Teff were stimulated with allogeneic cDC or pDC (1∶5, Teff:DC) in X-VIVO-15. After 7 days the induced cytokine profiles were investigated. Therefore, Teff were harvested and activated with 2.4 µg/ml PHA plus 1 ng/ml PMA for 5 h, monensin was added for last 4.5 hours. For intracellular analysis of cytokine production, anti-IL-2-PE, anti-IL-4-PE, anti-IL-10-PE, anti-IL-17A-PE, and anti-IFN-γ-FITC mAb, and FITC/PE-conjugated isotypic mAb (all BD Pharmingen) were used according to the manufacturer’s instructions.

For determination of IFN-α production, activated 10^6^/ml pDC (stimulated with CPG plus IL-3) and cDC (stimulated with the cytokine cocktail) were cultured for 24 h. Supernatants were harvested and IFN-α was detected by using a commercial available IFN-α ELISA (Diaclone, Besancon, France, detection limit 3.16 pg/ml) as indicated by the manufacturer.

### Statistical Analyses

Results represent means of triplicates ± standard deviation. Paired Student’s *t* test (two-tailed) was used for determination of statistical significance. Values of *p*<0.05 were considered significant and indicated in the corresponding figures (*: 0.01<*p*<0.05; **: 0.001<*p*<0.01; ***: *p*<0.001).

## Results

### Plasmacytoid Dendritic Cells are Weak Stimulators of T Effector Cells Compared to Conventional Dendritic Cells

Plasmacytoid DC represent approximately 0.1–0.3% of whole PBMC and can be distinguished upon expression of specific surface molecules. They are defined as lineage negative CD11c^−^ CD303^+^ CD304^+^ CD123^+^ cells and express low amounts of costimulatory molecules CD40, CD58, CD80 and CD86 in resting state (Fig. 1A).

For optimal activation, pDC were cultured with the TLR-9 ligand CpG in presence of IL-3. In this study, monocyte derived DC served as a well characterized standard for cDC, their activation was induced by a cocktail of proinflammatory cytokines [18]. Both activated DC subtypes showed comparable expression of CD86 and HLA-DR, other costimulatory molecules like CD40, CD58 and CD80 were expressed significantly higher on cDC (Fig. 1A). Therefore, cDC exhibited a strong capacity to induce proliferation and expansion of alloreactive CD4^+^ and CD8^+^ Teff (Fig. 1B and C). In line with their low expression of costimulatory molecules, activated pDC displayed a rather weak potential to activate and expand alloreactive Teff (Fig. 1B and C). Both DC populations were able to elicit production of effector cytokines in stimulated CD4^+^ Teff. While cDC stimulation resulted in strong production of both IL-2 and IFN-γ, CD4^+^ Teff cultured with activated pDC showed reduced levels of produced IL-2 and only weak IFN-γ secretion. At this time point, neither cDC- nor pDC-stimulation led to detectable production of IL-4, IL-10 or IL-17A (Fig. 1D).

### Absence of TREG ACTIVITY in Cocultures with Plasmacytoid Dendritic Cells

Treg-mediated suppression of Teff proliferation depends on the activation of Treg [10]. Here, we studied the functional properties of pDC and cDC in classical coculture suppressor assays. As control, we stimulated Teff and Treg with anti-CD3 mAb in presence of T cell-depleted PBMC. Under these conditions CD4^+^ and CD8^+^ Teff showed strong proliferation which was inhibited in coculture with Treg (Fig. 2A, right panels). Conventional DC with strong T cell stimulatory properties overcame Treg activity and induced full activation of CD4^+^ and CD8^+^ Teff even in cocultures with Treg. Surprisingly, pDC - weak T cell stimulators with low expression of costimulatory molecules – also allowed proliferation of CD4^+^ and CD8^+^ Teff in presence of Treg (Fig. 2A). Even the elevation of Treg frequencies in pDC-stimulated cocultures of Teff and Treg could not restore the suppression of Teff proliferation (Fig. 2B). As Treg function depends on their activation [10], we prestimulated Treg with either anti-CD3 mAb or pDC prior to applying them to CD4^+^ Teff. Teff proliferation was only suppressed if cocultured Treg were preactivated by anti-CD3 mAb stimulation, not after prestimulation with pDC (Fig. 2C), ruling out altered kinetics for pDC-mediated Treg-activation. Likewise, supplementation of pDC-stimulated cocultures with IL-2 or IFN-γ did not induce Treg-mediated suppression (Fig. 2D).

A hallmark of Treg is their anergic state *in vitro*, characterized by nonproliferation after T cell receptor-engaged stimulation. Since Tarbell et al. have shown that cDC overcome this anergy and induce Treg proliferation and expansion [13], we investigated the functional properties of pDC as Treg stimulators and analyzed the observed T cell proliferation in more detail. To determine Treg proliferation independent of proliferating Teff, Treg were labeled with the cell-tracing reagent eFluor®670. In control cultures, activated Treg showed no significant proliferation and remained anergic in cocultures with Teff. Conventional DC overcame Treg anergy and induced their proliferation and expansion, both in single and in coculture with Teff (Fig. 3A, middle row). In contrast, pDC did not break Treg anergy, neither in single culture nor in coculture with Teff (Fig. 3A, upper row). Underlining these results, in classical ^3^H-Thymidine-based proliferation assays we found Treg proliferation only after cDC stimulation, but not in response to pDC (Fig. 3B). Thus, in pDC-stimulated cocultures, proliferation is restricted to Teff, while Treg remain nonproliferative but incapable to suppress Teff activation.

### Blockade of Proinflammatory Cytokines does not Restore Treg Function in Presence of Plasmacytoid DC

Plasmacytoid DC are strong producers of type-I interferons after activation with CpG (Fig. 4A, n.d.: not detectable) whereas cDC produce high amounts of proinflammatory cytokines such as TNF-α or IL-6 but not IFN-α (Fig. 4A, n.d.: not detectable). To investigate if the proinflammatory cytokines IL-1β, IL-6, TNF-α and IFN-α were responsible for the absence of suppressive Treg activity in cocultures with pDC, we added neutralizing mAb to these cultures. However, neither blockade of intrinsic IFN-α nor neutralization of the proinflammatory cytokines IL-6 or TNF-α restored suppressive Treg activity. Only neutralization of IL-1β slightly reduced Teff proliferation (Fig. 4B). Thus, we assume that pDC overcome Treg-mediated suppression independent of these proinflammatory cytokines. To exclude that soluble mediators required for suppression are produced after polyclonal but not after pDC-stimulation, supernatants from polyclonal stimulated cocultures were added to pDC-stimulated cocultures. As addition of these supernatants did not induce suppression in pDC-stimulated cocultures, we conclude that absence of suppression is not due to lack of soluble mediators (Fig. 4C).

### Plasmacytoid DC Ineffectively Activate Foxp3^+^ Regulatory T Cells

Functional activation of resting Treg is associated with upregulation of distinct surface molecules. For example, Treg-mediated suppression requires surface expression of cytotoxic T lymphocyte antigen-4 (CTLA-4) [19], which is detectable on Treg after sufficient functional activation [10]. More recently, glycoprotein-A repetitions predominant (GARP) was identified as a Treg surface activation marker, important for their suppressive function [20]. To determine the activation status of Treg after stimulation with pDC, we analyzed their extracellular CTLA-4 and GARP expression before and after activation. As shown in Fig. 5A, both molecules were not detectable on the surface of resting Treg. After polyclonal activation CTLA-4 and GARP were upregulated. Comparable upregulation was observed after stimulation with cDC. In contrast, only very few pDC-stimulated Treg, expressed CTLA-4 or GARP on the surface. Since these results suggested that pDC are inefficient activators of human Treg, we investigated whether additional stimulatory signals could increase CTLA-4 and GARP surface expression in presence of pDC. Whereas costimulation with anti-CD28 mAb showed no effect (not shown) anti-CD3 mAb induced GARP and CTLA-4 expression on pDC-stimulated Treg (Fig. 5A, right panels). Therefore, we presumed that an ineffective Treg stimulation by pDC could be responsible for the absence of suppressive activity in pDC-stimulated Treg cultures.

To evaluate our thesis, we added either anti-CD3 or anti-CD28 mAb to cocultures of Teff, Treg and allogeneic pDC. Additionally, we used anti-CD40L stimulation in these assays since pDC expressed significantly less CD40 than cDC. Increased costimulation with anti-CD28 or anti-CD40L mAb did not rebuild Treg-mediated Teff suppression (Fig. 5B). However, anti-CD3 stimulation, a well-known signal for functional activation of Treg [33] in addition to pDC stimulation restored suppressive Treg function in a dose-dependent manner, resulting in inhibited Teff proliferation (Fig. 5B). Interestingly, blockade of CTLA-4 abrogated the induced suppression, while GARP-blockade was less effective (Fig. 5C). Therefore, we conclude that the overall stimulatory potential of pDC is insufficient for functional activation of Treg but efficient enough to stimulate Teff proliferation. Strong CD3-crosslinking by anti-CD3 mAb can compensate for inadequate pDC stimulation and induce suppressive Treg function.

Thus, our data demonstrate that pDC as well as cDC induce reasonable Teff proliferation in presence of stimulated Treg. But in contrast to cDC, pDC are inefficient in induction of Treg proliferation. Therefore, pDC stimulation allows the expansion of Teff without bystander activation and proliferation of Treg.

## Discussion

In this study we compared the stimulatory properties of human pDC and cDC in cocultures with Teff and Treg. Our data demonstrate that pDC deliver insufficient stimulation for functional activation of Treg. On the other hand, stimulatory capacity of pDC is sufficient to induce Teff activation and expansion. Accordingly, pDC-activated human Treg are incapable to suppress Teff proliferation. In contrast to pDC, cDC break Treg anergy, abrogate their suppressive activity and simultaneously induce Teff and Treg proliferation, as shown before [12], [21].

Functional properties of cDC and pDC depend on their activation by individual inflammatory agents present in the local milieu. Both DC subsets showed a strong upregulation of costimulatory molecules upon activation, as shown previously [2]. However, cDC acquired significant higher expression of costimulatory molecules such as CD40, CD80 and CD58. Consequently, cDC exhibited stronger capacities to induce full activation and expansion of CD4^+^ and CD8^+^ Teff, while pDC are poor Teff stimulators. This is in line with many studies from other groups, demonstrating weak T cell stimulation by pDC [4], [22]–[24]. Aside from their immunogenic functions, cDC [3] and also pDC [7] contribute to induction of tolerance. It has been shown that activated pDC often favor the differentiation of naïve CD4^+^ and CD8^+^ T cells [7], [37] into induced Treg [4], [6].

Functional consequences of pDC stimulation on the suppressive capacity of human naturally occurring Foxp3^+^ Treg have never been addressed. Ouabed et al. demonstrated that splenic CpG-activated pDC from rats are able to evoke proliferative responses in CD4^+^CD25^+^ Treg. They further showed that this pDC-induced Treg proliferation was crucial for their loss of suppressive capacity [25]. In contrast, as shown here human pDC-stimulated Treg remained anergic but pDC still elicit Teff proliferation in presence of Treg. We suggest that the observed abrogation of Treg function in pDC-stimulated cocultures was due to inefficient Treg activation and not to inhibition of Treg function as additional anti-CD3 mAb stimulation partially restores suppressive capacity. The lack of human Treg proliferation can be explained either by different functional properties of human versus rat pDC or rather by additional inflammatory mediators produced in the spleen of rat during CpG-stimulation that costimulate pDC-induced Treg activation *in vivo*. We observed Treg expansion only in cDC-stimulated cultures, as described earlier by Tarbell et al. and others [13], [14], [26].

The here presented findings are of special interest, insofar as human pDC have been connected to the pathogenesis of certain autoimmune diseases [27], [28]. Circumvention of Treg-mediated suppression and activation of autoreactive T cells by pDC might precise the involvement of pDC in autoimmunity. Several groups reported that activation of pDC, followed by production of type-I interferons in absence of infection plays a role in the development of autoimmune diseases. Using a xenograft model of human psoriasis Nestle et al. showed that pDC migrate into the skin of psoriatic patients. Here, production of IFN-α upon pDC activation accelerated the disease [29]. Furthermore, pDC-derived IFN-α was suggested to correlate with disease severity in patients of systemic lupus erythematosus [30]–[32]. However, in the latter IFN-α was dispensable for the absence of Treg function. Teff proliferation in presence of Treg was independent of IL-6, a proinflammatory cytokine proposed to modulate Treg function, at least in case of LPS-stimulated murine bone marrow derived DC [12]. Only neutralization of IL-1β partially inhibited Teff proliferation. However, IL-1β is a Teff costimulatory cytokine and not produced by pDC [1]. Therefore, the observed effect appears to be Teff intrinsic and not mediated by pDC. Besides cell contact-dependent mechanisms it is described that suppression by Treg can also be mediated by soluble factors [8]. Supernatants of standard suppressor cultures which include all these potential factors did not restore suppression in pDC-stimulated cocultures. As demonstrated by de la Rosa et al., Treg function depends on IL-2. [40]. Furthermore, Sawitzki et al. showed the importance of IFN-γ for Treg function [41]. Insufficient IL-2 or IFN-γ production in pDC-stimulated cultures as reasons for absence of Treg function was excluded since exogenous IL-2 and IFN-γ could not restore Treg-mediated suppression.

For suppressive capacity, Treg require an adequate stimulation [10], [33]. Human Treg activation and function associates with the upregulation of the surface molecules CTLA-4 and GARP [10], [20], we therefore analyzed the expression of both molecules. In line with the absence of suppressive activity, only weak expression of CTLA-4 and GARP on very few pDC-stimulated Treg was detectable. This supported our thesis that pDC delivered inadequate Treg activating signals. Strong antibody-mediated CD3 crosslinking induced upregulation of both markers and enabled Treg function in presence of pDC, whereas extension of costimulation did not. T cell activation is an integrative process of TCR-dependent and independent signals [34]. Activated pDC and cDC expressed comparable amounts of HLA-DR and both DC were able to induce proliferation of alloreactive CD4^+^ Teff. Therefore, the TCR signal provided by pDC is in general strong enough to induce T cell activation but the total signal given by pDC is too weak to activate Treg function. This might also explain why human Treg modulate only function of cDC but not of pDC [35] since inactivated Treg have no effector function.

Several groups suggested the ability of human pDC to convey suppressive activity *de novo* in CD4^+^ T helper cells as well as in CD8^+^ T cells [4], [36], [37]. On the opposite, pDC are essentially involved in protective anti-viral immunity, priming of melanoma-specific CD8^+^ T cells and the pathogenesis of several diseases [16], [38], [39]. Thus, pDC are inevitable for maintenance of tolerance and protective immunity. However, the impact of pDC on individual immune responses is still controversial and probably depends on their activation state, distribution and migration patterns.

Here we describe a new function of pDC. By providing stimulatory signals sufficient for Teff proliferation, but insufficient for functional Treg activation, pDC circumvent the suppressive activity of naturally occurring human Treg. Thus, we suggest that pDC do not reach the essential signal strength Treg require for their functional activation, resulting in anergic Treg without suppressive activity.

## References

[1] Liu YJ (2005) IPC: professional type 1 interferon-producing cells and plasmacytoid dendritic cell precursors. Annu Rev Immunol 23: 275–306. 15771572 10.1146/annurev.immunol.23.021704.115633

[2] Banchereau J, Steinman RM (1998) Dendritic cells and the control of immunity. Nature 392: 245–252. 9521319 10.1038/32588

[3] Jonuleit H, Schmitt E, Schuler G, Knop J, Enk AH (2000) Induction of interleukin 10-producing, nonproliferating CD4(+) T cells with regulatory properties by repetitive stimulation with allogeneic immature human dendritic cells. J Exp Med 192: 1213–1222. 11067871 10.1084/jem.192.9.1213 PMC2193357

[4] Moseman EA, Liang X, Dawson AJ, Panoskaltsis-Mortari A, Krieg AM, et al (2004) Human plasmacytoid dendritic cells activated by CpG oligodeoxynucleotides induce the generation of CD4+CD25+ regulatory T cells. J Immunol 173: 4433–4442. 15383574 10.4049/jimmunol.173.7.4433

[5] Hanabuchi S, Ito T, Park WR, Watanabe N, Shaw JL, et al (2010) Thymic stromal lymphopoietin-activated plasmacytoid dendritic cells induce the generation of FOXP3+ regulatory T cells in human thymus. J Immunol 184: 2999–3007. 20173030 10.4049/jimmunol.0804106 PMC3325785

[6] Martin-Gayo E, Sierra-Filardi E, Corbi AL, Toribio ML (2010) Plasmacytoid dendritic cells resident in human thymus drive natural Treg cell development. Blood 115: 5366–5375. 20357241 10.1182/blood-2009-10-248260

[7] Matta BM, Castellaneta A, Thomson AW (2010) Tolerogenic plasmacytoid DC. Eur J Immunol 40: 2667–2676. 20821731 10.1002/eji.201040839 PMC3974856

[8] Sakaguchi S, Miyara M, Costantino CM, Hafler DA (2010) FOXP3+ regulatory T cells in the human immune system. Nat Rev Immunol 10: 490–500. 20559327 10.1038/nri2785

[9] Sakaguchi S (2005) Naturally arising Foxp3-expressing CD25+CD4+ regulatory T cells in immunological tolerance to self and non-self. Nat Immunol 6: 345–352. 15785760 10.1038/ni1178

[10] Jonuleit H, Schmitt E, Stassen M, Tuettenberg A, Knop J, et al (2001) Identification and functional characterization of human CD4(+)CD25(+) T cells with regulatory properties isolated from peripheral blood. J Exp Med 193: 1285–1294. 11390435 10.1084/jem.193.11.1285 PMC2193380

[11] Jonuleit H, Schmitt E (2003) The regulatory T cell family: distinct subsets and their interrelations. J Immunol 171: 6323–6327. 14662827 10.4049/jimmunol.171.12.6323

[12] Pasare C, Medzhitov R (2003) Toll pathway-dependent blockade of CD4+CD25+ T cell-mediated suppression by dendritic cells. Science 299: 1033–1036. 12532024 10.1126/science.1078231

[13] Tarbell KV, Petit L, Zuo X, Toy P, Luo X, et al (2007) Dendritic cell-expanded, islet-specific CD4+ CD25+ CD62L+ regulatory T cells restore normoglycemia in diabetic NOD mice. J Exp Med 204: 191–201. 17210729 10.1084/jem.20061631 PMC2118426

[14] Yamazaki S, Iyoda T, Tarbell K, Olson K, Velinzon K, et al (2003) Direct expansion of functional CD25+ CD4+ regulatory T cells by antigen-processing dendritic cells. J Exp Med 198: 235–247. 12874257 10.1084/jem.20030422 PMC2194081

[15] Lande R, Gilliet M (2010) Plasmacytoid dendritic cells: key players in the initiation and regulation of immune responses. Ann N Y Acad Sci 1183: 89–103. 20146710 10.1111/j.1749-6632.2009.05152.x

[16] Swiecki M, Colonna M (2010) Accumulation of plasmacytoid DC: Roles in disease pathogenesis and targets for immunotherapy. Eur J Immunol 40: 2094–2098. 20853492 10.1002/eji.201040602 PMC3732170

[17] Becker C, Taube C, Bopp T, Becker C, Michel K, et al (2009) Protection from graft-versus-host disease by HIV-1 envelope protein gp120-mediated activation of human CD4+CD25+ regulatory T cells. Blood 114: 1263–1269. 19439734 10.1182/blood-2009-02-206730

[18] Jonuleit H, Kuhn U, Muller G, Steinbrink K, Paragnik L, et al (1997) Pro-inflammatory cytokines and prostaglandins induce maturation of potent immunostimulatory dendritic cells under fetal calf serum-free conditions. Eur J Immunol 27: 3135–3142. 9464798 10.1002/eji.1830271209

[19] Tang Q, Boden EK, Henriksen KJ, Bour-Jordan H, Bi M, et al (2004) Distinct roles of CTLA-4 and TGF-beta in CD4+CD25+ regulatory T cell function. Eur J Immunol 34: 2996–3005. 15468055 10.1002/eji.200425143

[20] Wang R, Kozhaya L, Mercer F, Khaitan A, Fujii H, et al (2009) Expression of GARP selectively identifies activated human FOXP3+ regulatory T cells. Proc Natl Acad Sci U S A 106: 13439–13444. 19666573 10.1073/pnas.0901965106 PMC2726405

[21] Banerjee DK, Dhodapkar MV, Matayeva E, Steinman RM, Dhodapkar KM (2006) Expansion of FOXP3high regulatory T cells by human dendritic cells (DCs) in vitro and after injection of cytokine-matured DCs in myeloma patients. Blood 108: 2655–2661. 16763205 10.1182/blood-2006-03-011353 PMC1895594

[22] Boonstra A, Asselin-Paturel C, Gilliet M, Crain C, Trinchieri G, et al (2003) Flexibility of mouse classical and plasmacytoid-derived dendritic cells in directing T helper type 1 and 2 cell development: dependency on antigen dose and differential toll-like receptor ligation. J Exp Med 197: 101–109. 12515817 10.1084/jem.20021908 PMC2193804

[23] Grouard G, Rissoan MC, Filgueira L, Durand I, Banchereau J, et al (1997) The enigmatic plasmacytoid T cells develop into dendritic cells with interleukin (IL)-3 and CD40-ligand. J Exp Med 185: 1101–1111. 9091583 10.1084/jem.185.6.1101 PMC2196227

[24] Cella M, Facchetti F, Lanzavecchia A, Colonna M (2000) Plasmacytoid dendritic cells activated by influenza virus and CD40L drive a potent TH1 polarization. Nat Immunol 1: 305–310. 11017101 10.1038/79747

[25] Ouabed A, Hubert FX, Chabannes D, Gautreau L, Heslan M, et al (2008) Differential control of T regulatory cell proliferation and suppressive activity by mature plasmacytoid versus conventional spleen dendritic cells. J Immunol 180: 5862–5870. 18424705 10.4049/jimmunol.180.9.5862

[26] Yamazaki S, Patel M, Harper A, Bonito A, Fukuyama H, et al (2006) Effective expansion of alloantigen-specific Foxp3+ CD25+ CD4+ regulatory T cells by dendritic cells during the mixed leukocyte reaction. Proc Natl Acad Sci U S A 103: 2758–2763. 16473944 10.1073/pnas.0510606103 PMC1413800

[27] Chen SC, de Groot M, Kinsley D, Laverty M, McClanahan T, et al (2010) Expression of chemokine receptor CXCR3 by lymphocytes and plasmacytoid dendritic cells in human psoriatic lesions. Arch Dermatol Res 302: 113–123. 19517126 10.1007/s00403-009-0966-2

[28] Gilliet M, Cao W, Liu YJ (2008) Plasmacytoid dendritic cells: sensing nucleic acids in viral infection and autoimmune diseases. Nat Rev Immunol 8: 594–606. 18641647 10.1038/nri2358

[29] Nestle FO, Conrad C, Tun-Kyi A, Homey B, Gombert M, et al (2005) Plasmacytoid predendritic cells initiate psoriasis through interferon-alpha production. J Exp Med 202: 135–143. 15998792 10.1084/jem.20050500 PMC2212894

[30] Han GM, Chen SL, Shen N, Ye S, Bao CD, et al (2003) Analysis of gene expression profiles in human systemic lupus erythematosus using oligonucleotide microarray. Genes Immun 4: 177–186. 12700592 10.1038/sj.gene.6363966

[31] Bennett L, Palucka AK, Arce E, Cantrell V, Borvak J, et al (2003) Interferon and granulopoiesis signatures in systemic lupus erythematosus blood. J Exp Med 197: 711–723. 12642603 10.1084/jem.20021553 PMC2193846

[32] Farkas L, Beiske K, Lund-Johansen F, Brandtzaeg P, Jahnsen FL (2001) Plasmacytoid dendritic cells (natural interferon- alpha/beta-producing cells) accumulate in cutaneous lupus erythematosus lesions. Am J Pathol 159: 237–243. 11438470 10.1016/s0002-9440(10)61689-6 PMC1850412

[33] Thornton AM, Shevach EM (1998) CD4+CD25+ immunoregulatory T cells suppress polyclonal T cell activation in vitro by inhibiting interleukin 2 production. J Exp Med 188: 287–296. 9670041 10.1084/jem.188.2.287 PMC2212461

[34] Noel G, Brinster C, Semana G, Bruniquel D (2009) Modulation of the TCR stimulation strength can render human activated CD4+ T cells suppressive. Int Immunol 21: 1025–1036. 19625380 10.1093/intimm/dxp068

[35] Houot R, Perrot I, Garcia E, Durand I, Lebecque S (2006) Human CD4+CD25high regulatory T cells modulate myeloid but not plasmacytoid dendritic cells activation. J Immunol 176: 5293–5298. 16621995 10.4049/jimmunol.176.9.5293

[36] Ito T, Yang M, Wang YH, Lande R, Gregorio J, et al (2007) Plasmacytoid dendritic cells prime IL-10-producing T regulatory cells by inducible costimulator ligand. J Exp Med 204: 105–115. 17200410 10.1084/jem.20061660 PMC2118437

[37] Boor PPC, Metselaar HJ, de Jonge S, Mancham S, van der Laan LJW, et al (2011) Human plasmacytoid dendritic cells induce CD8^+^ LAG-3^+^Foxp3^+^CTLA-4^+^ regulatory T cells that suppress allo-reactive memory T cells. Eur J Immunol 41: 1663–1674. 21469126 10.1002/eji.201041229

[38] Takagi H, Fukaya T, Eizumi K, Sato Y, Sato K, et al (2011) Plasmacytoid dendritic cells are crucial for the initiation of inflammation and T cell immunity in vivo. Immunity 35: 958–971. 22177923 10.1016/j.immuni.2011.10.014

[39] Salio M, Cella M, Vermi W, Facchetti F, Palmowski MJ, et al (2003) Plasmacytoid dendritic cells prime IFN-gamma-secreting melanoma-specific CD8 lymphocytes and are found in primary melanoma lesions. Eur J Immunol 33: 1052–1062. 12672071 10.1002/eji.200323676

[40] de la Rosa M, Rutz S, Dorninger H, Scheffold A (2004) Interleukin-2 is essential for CD4+CD25+ regulatory T cell function. Eur J Immunol 34: 2480–2488. 15307180 10.1002/eji.200425274

[41] Sawitzki B, Kingsley CI, Olivieira V, Karim M, Herber M, et al (2005) IFN-g production by alloantigen-reactive regulatory T cells is important for their regulatory function in vivo. J Exp Med 12: 1925–1935. 10.1084/jem.20050419 PMC2212028 15967822

